# Larval cases of caddisfly (Insecta: Trichoptera) affinity in Early Permian marine environments of Gondwana

**DOI:** 10.1038/srep19215

**Published:** 2016-01-14

**Authors:** Lucas D. Mouro, Michał Zatoń, Antonio C.S. Fernandes, Breno L. Waichel

**Affiliations:** 1 Programa de Formação de Recursos Humanos, PFRH-PB 240,Universidade Federal de Santa Catarina, 88040-900, Brazil; 2 Department of Palaeontology & Stratigraphy, Faculty of Earth Sciences, University of Silesia, 41-200 Sosnowiec, Poland; 3 Museu Nacional, Universidade Federal do Rio de Janeiro, 20940-040, Brazil; 4 Programa de Pós-Graduação em Geologia, Universidade Federal do Rio de Janeiro, 21.941-916, Brazil

## Abstract

Caddisflies (Trichoptera) are small, cosmopolitan insects closely related to the Lepidoptera (moths and butterflies). Most caddisflies construct protective cases during their larval development. Although the earliest recognisable caddisflies date back to the early Mesozoic (Early and Middle Triassic), being particularly numerous and diverse during the Late Jurassic and Early Cretaceous, the first records of their larval case constructions are known exclusively from much younger, Early to Middle Jurassic non-marine deposits in the northern hemisphere. Here we present fossils from the Early Permian (Asselian–Sakmarian) marine deposits of Brazil which have strong morphological and compositional similarity to larval cases of caddisflies. If they are, which is very probable, these finds not only push back the fossil record of true caddisflies, but also indicate that their larvae constructed cases at the very beginning of their evolution in marine environments. Since modern caddisflies that construct larval cases in marine environments are only known from eastern Australia and New Zealand, we suggest that this marine ecology may have first evolved in western Gondwana during the Early Permian and later spread across southern Pangea.

Caddisflies (order Trichoptera) are small, holometabolous (having a pupal stage in their developmental cycle) insects closely related to Lepidoptera (moths and butterflies)^1^,^2^. The group comprises more than 12000 living species known from nearly all over the world, and nearly 500 fossil species^2^. Most caddisflies (Integripalpia group) construct cases during larval development in aquatic (usually freshwater) environments. Caddisfly larvae (or caddis worms) construct their cases using secreted silk^1^,^3^,^4^,^5^, as well as selected foreign components, such as sand grains, mollusk shells or plant fragments^1^,^2^,^3^,^6^. This behavior probably has mainly an anti-predatory purpose with the cases providing mechanical defense and camouflage^4^,^7^. However, such behaviour also has a considerable advantage for reproductive success and food gathering^3^ as well as for respiration by generating currents through the case via abdominal ventilation^8^,^9^. Experimental studies have shown that caddisfly larvae can select particles and build their cases achieving maximal energetic gain with minimal energy expenditure^3^. The selection of a variety of particles by different species for case construction results in an enormous number of different kinds of cases^1^,^6^,^10^,^11^. Thus, the ability of larval trichopterids to construct cases from silk and surrounding materials has led to their ecological diversification and utilization of habitats unavailable to other aquatic macroinvertebrates.

Being constructed of tough and durable components, caddisfly larval cases fossilized much easier and more often than the associated larvae whose bodies are covered by decay-prone, chitinous exoskeletons. Thus, the fossil record of caddisflies consists mainly of larval cases, while the body fossils of their producers are extremely rare^6^,^12^. There is a discrepancy in appearance of adult caddisfly body fossils and larval cases in the fossil record. While the oldest body fossils of true caddisflies are known from the Early and Middle Triassic (ca. 230 million years ago)^1^,^2^,^13^, being particularly numerous and diverse during the Late Jurassic and Early Cretaceous^1^,^6^,^9^,^11^,^14^, the oldest larval cases are known from Early to Middle Jurassic^1^,^15^,^16^. Importantly, all fossil caddis worm cases thus far reported come from non-marine deposits.

Here we report on intriguing fossils found in the Lower Permian (Asselian–Sakmarian) marine deposits of southern Brazil, which are very similar to caddisfly larval cases. As these caddisfly-like larval cases are the oldest reported so far and come from a marine palaeoenvironment of the Gondwanan sector of the Pangea supercontinent, they would not only push back the fossil record of true caddisflies, but also shed a new light on ecology and behaviour at their very early stages of evolution.

## Results

### Stratigraphy and palaeoenvironment

**Figure 1:**
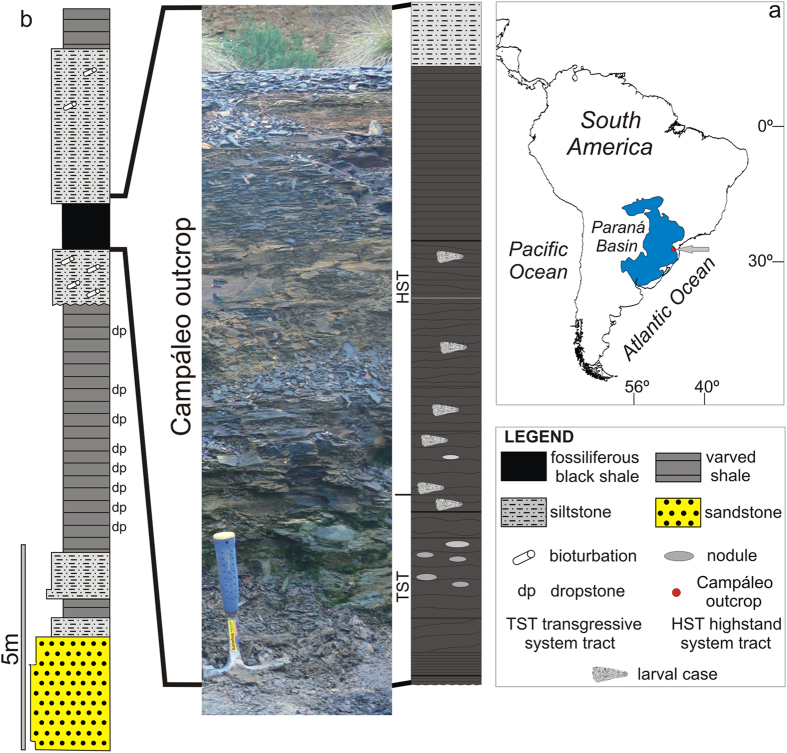
Locality and lithology. (**a**) Locality of the Campáleo outcrop in Brazil (arrowed). (**b**) Lithological section of the Lower Permian Lontras Shale (left) with the fossiliferous black shales containing the larval cases at the Campáleo site (right). The map was drawn using Corel Draw x5.

The fossils reported here were found in the Campáleo outcrop (S 26°09′30.22″, W 49°48′52.82″) located in the city of Mafra, State of Santa Catarina, southern Brazil (Fig. 1a, see also Supplementary Fig. 1). The section comprises sedimentary rocks deposited in the eastern part of the Paraná Basin during a post-glacial marine transgressive event, consisting of varved shale with dropstones, bioturbated and laminated siltstones, and fossiliferous black shale^17^. The section (Fig. 1b) is a part of the Lontras Shale, which is the upper part of the Campo Mourão Formation, dated as Lower Permian (Asselian-Sakmarian)^18^. This age is supported by palynological dating which indicates the *Protohaploxypinus goraiensis* Subzone of the*Vittatina costabilis* Interval Zone (Asselian-Sakmarian)^19^. The cases were collected in the 1.1 m thick fossiliferous black shale that also contains hexactinellid sponges, brachiopods, gastropods, bivalves, crustaceans, actinopterygian fishes, coelacanth scales, shark teeth, conodont elements, scolecodonts, insects, as well as coprolites and plant fragments^20^,^21^,^22^,^23^,^24^,^25^,^26^,^27^. Such a fossil assemblage, and especially the presence of terrestrial elements such as sporomorphs, plant remains and insects, point to a marine environment close to land. The presence of abundant amorphous organic matter (AOM), prasinophytes (*Tasmanites*), conodonts and autochthonous hexactinellid sponges in the lower 35 cm of the section point to a calm, deeper, subtidal palaeoenvironment. Higher up in the section the contribution of AOM decreases and phytoclasts become more abundant indicating a marine palaeoenvironment more proximal to the land^25^. The presence of framboid pyrite disseminated in the host deposits points to at least periodic oxygen-deficient bottom waters during deposition of the shales. The Campáleo site is the only place where fossiliferous, case-bearing deposits of the Lontras Shale crop out^26^.

### Characteristics of the cases and their preservation

**Figure 2:**
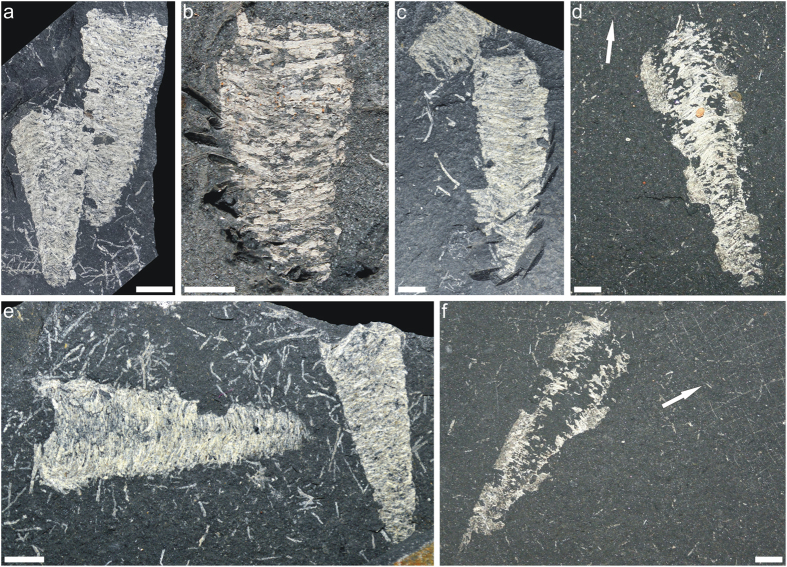
Early Permian fossils interpreted as caddisfly larval cases from Campáleo outcrop, Brazil. (**a,c,e**) Close association of two larval cases surrounded by dispersed silk strips. (**b,d,f**) Single individuals, one with well-visible silk strips (**b**) and two (**d,f**) associated with hexactinellid sponge skeletons (arrowed). (**a**) CPE 5917, (**b**) CPI 454, (**c**) CPE 7812, (**d**) CPI 679, (**e**) CPE 7811, (**f**) CPI 680. Scale bars 5 mm.

The fossils interpreted as caddisfly larval cases are flattened, cylindrical, distinctly tapering to the insect posterior (Fig. 2). However, the more or less flat tops and bases suggest that both ends of the cases were opened. The size of the cases varies widely. Their heights range from 13 mm to 52 mm (mean = 29.5 mm, st. dev. = 9.11, *n* = 83), and their widths range from 7 mm to 25 mm (mean = 14.1 mm, st. dev. = 3.47, *n* = 83). Usually, they are preserved on the bedding planes as single individuals, but a close association of a couple of cases also occur (Fig. 2a,c,e).

**Figure 3:**
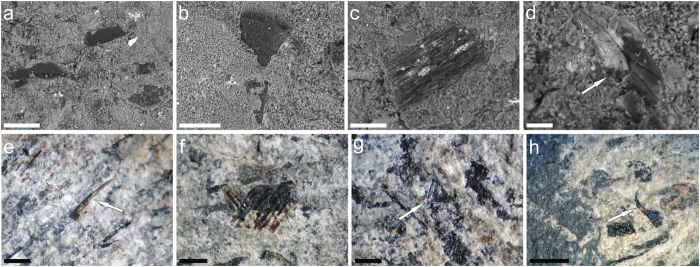
Foreign particles gathered by the larvae and adhered to the silk cases. (**a–c**) Plant fragments, CPE 3142. (**d**) insect fragment (arrowed), CPE 3142. (**e**) fish tooth, CPE 5917. (**f**) fish scales, CPE 3089. (**g**) sponge spicule (arrowed), CPE 5919. (**h**) scolecodont (arrowed), CPI 124. ESEM photomicrographs (**a–d**) and binocular microscope photomicrographs (**e–h**). Scale bars, 100 μm (**a–b**), 50 μm (**c**), 20 μm (**d**), 500 μm (**e–g**), 10 μm (**h**).

The cases are primarily composed of whitish, transversely arranged and tightly joined together thin strips (Fig. 2 and Supplementary Fig. 3). In some instances, however, the cases may be completely disintegrated in the form of isolated strips chaotically scattered on the bedding plane (Fig. 2a,e; Supplementary Fig. 3b). The strips form the main part of the cases and presumably represent the fossilized remnants of the silk material used by the larvae for case construction. Plant fragments varying in shape and size are usually randomly adhered to the silk (Fig. 3a–c); however, some plant fragments may be transversely attached. Sometimes other particles, such as fish scales and teeth, scolecodonts (annelid jaw elements), sponge spicules and insect remains, are also attached to the strips (Fig. 3d–h).

**Figure 4:**
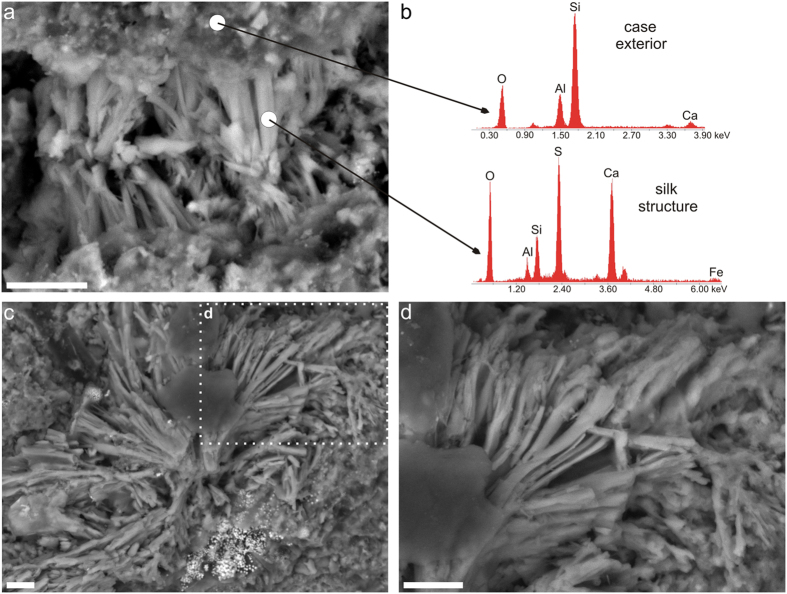
Structure and composition of the Early Permian larval cases. (**a,c,d**) ESEM photomicrographs of thread-like structures of the silk building the larval cases. (**b**) EDS spectra showing the elemental composition of the case exterior and silk structure. White circles on (**a**) indicate the spots of EDS analyses. Scale bars 10 μm.

EDS analysis showed that the exterior of the silk material is covered by aluminosilicates (62.64%) (Fig. 4b). The preserved silk material has a distinct, thread-like structure as suggested by exfoliated parts of the cases (Fig. 4a,c,d). This structure is quite similar to silk produced by Recent trichopteran larvae^4^. EDS showed that it is distinctly enriched in sulphur and calcium, and thus completely different in composition from its exterior surface (Fig. 4b).

## Discussion

The fossils reported here are unique and readily distinguishable from both other body fossils having similar, cone-shaped morphologies and protective structures constructed by other aquatic animals. The general appearance and composition clearly distinguish the fossils from the associated hexactinellid sponges. With respect to composition and external characteristics (the presence of strips with adhering foreign particles instead of distinct growth lines, striae or ridges), the fossils reported are also easily distinguished from both any thecae of animals with cnidarian affinity, like conulariids^28^, tubes of cnidarian-like fossils similar to such taxa as *Byronia*^29^, and cone-shaped tubes of priapulid worms as *Selkirkia*^30^. The morphology of the narrow posterior end of the fossils discussed here also indicates that it was not an attachment base like that present in such cnidarian-like fossils as conulariids or *Byronia* and related forms.

One may assume that similar cases are produced by the polychaete family, Pectinariidae. Indeed, pectinariid tubes are also straight, conical and tapering towards its base and made of detrital and sand particles^31^. Similar agglutinated tubes are also known from some Palaeozoic and Mesozoic deposits^32^,^33^,^34^. However, unlike the pectinariid polychaetes or other agglutinated tubes known from the fossil record, they are mainly composed of silk-like material with some addition of other particles gathered from the sea-bottom. Moreover, the cases described here occur in fine-grained siliciclastics, while the modern pectinariids live on sandy substrates that provide the building material^11^,^31^. The cases described may contain fish scales and teeth, particles not used by modern agglutinating polychaetes but known to occur in other fossil caddisfly larval cases^11^, and they are very similar or even identical to some other caddis worm cases known from non-marine deposits, especially those from the Middle Jurassic of Inner Mongolia^16^. Moreover, the silk forming the larval cases possesses characteristic thread-like structure (Fig. 4c,d) and thus is very similar to that present in the Recent forms^4^,^35^. Aluminosilicates present on the surface of the silk strips, as suggested by the EDS analysis, most likely originated from the clay-rich shales, since the silk interior has a completely different composition, being enriched in sulphur and calcium and containing fewer aluminosilicates. Interestingly, the silk of modern caddis worm cases is also enriched in calcium and sulphur elements^4^. Thus, all the features outlined above strongly support the caddisfly affinity of the cases studied.

Both the closely-related orders, Trichoptera and Lepidoptera, form the lineage Amphiesmenoptera^2^,^36^,^37^. Due to an overall morphological similarity, the taxonomic boundary between Trichoptera and Lepidoptera is obscured in the early fossil record^37^. Although the earliest unequivocal lepidopteran is Early Jurassic in age^36^,^37^, there is uncertainty concerning the nature and the first appearance of the oldest caddisflies. Representatives of the suborder Protomeropina (=Permotrichoptera, a stem-group Amphiesmenoptera) were considered either as the oldest caddisflies, their close relatives, or their ‘ancestors’^1^,^36^,^38^,^39^. If we assume Protomeropina as Trichoptera, then their earliest fossil record in the northern hemisphere (Czech Republic and Kansas, USA) is dated to the Artinskian (mid-Early Permian)^1^, and in the southern hemisphere (South Africa and Australia) they are known from the Upper Permian^1^,^38^. However, if we exclude Protomeropina representatives from Trichoptera or the direct lineage leading to the group, then the earliest records of recognisable caddisflies come from the Early and Middle Triassic^1^,^2^,^13^. Whatever the consensus, all the records mentioned above are represented by body fossils of the adults, while the caddisfly larval cases are only known since the Early and Middle Jurassic of northern hemisphere: Transbaikalia and Inner Mongolia^1^,^15^,^16^. Thus, if the larval cases reported here are trichopteran, then they not only point to the oldest record of true caddisflies known so far, but also to the earliest evidence of their case-making behaviour. The cases reported here, however, do not necessarily indicate that caddisflies originated in Gondwana during the Early Permian, because the true holometabolous insects (to which the caddisflies belong) are known from the Carboniferous of the northern hemisphere (France)^39^. However, this find would also indicate that there was no significant time-gap between the appearance of caddisflies in general and the behaviour of case-building by their larvae. This would be a rapid innovation in caddisfly evolution that probably appeared shortly after they originated.

The particles attached to the silk cases are mainly represented by plant fragments. Although some are transversely aligned, the majority have various shapes that indicate the larvae did not spend time on careful preparation (cutting) of the material, but collected the plant detritus randomly from the sea-bottom. The Early Permian larvae also did not select the material for case construction, as apart of plant material, they also gathered other particles present in the environment, such as fish scales and teeth, scolecodonts, sponge spicules and insect fragments. The silk cases and unsorted, randomly selected particles are considered as the simplest of constructions^1^ and those discussed here are evidence that such behaviour was characteristic of these larval case builders. The Early Permian larvae were at the first stages of behavioural evolution in case construction. Later in their evolution, the larvae were able to construct more durable cases made of specific particles consisting solely of, for example, plant remains, shell fragments, mineral grains or fish bones and scales. Such sophisticated larval cases built by carefully prepared and selected material are not known before the Middle Jurassic^1^,^11^,^15^. However, it must be stressed that evolution of caddisflies is not parallel with the behavioural evolution of their larvae, as constructionally simpler larval cases have not been directly replaced by more sophisticated ones; instead, they were still produced alongside modern cases^1^.

Another significant point of the present discovery is the ecological evolution of caddisflies. Until now, the body fossils, as well as larval cases, were found exclusively in non-marine palaeoenvironments^1^,^40^. This may confirm the general evolutionary pathway of the group, as most Recent species occupy freshwater settings. Although some fossil caddisfly cases were reported from deposits indicating saline lake palaeoenvironments^41^,^42^, only a few species of Recent caddisflies occupy normal-marine waters. These are known only from Australia and New Zealand^43^,^44^,^45^,^46^. The Early Permian larval cases reported here, however, indicate that larvae of caddisfly ancestors occupied and built the cases in marine habitats very early in their evolutionary history. Living in marine waters, the larvae must have possessed sufficient osmoregulation capacity to maintain the osmotic pressure of their haemolymph at a constant level. In such a marine environment, for the caddisfly species, *Philanisus plebeius*, drinking the medium is an important feature of its osmoregulatory mechanism. Moreover, this mechanism only operates efficiently if the larva sits inside its case. When it is outside, it loses its weight rapidly due to the great reduction in the rate of drinking^45^. Thus, the caddisfly larval case in a marine environment has an additional, important advantage which may have evolved very early in the evolutionary history of the group. This significantly changes our view of their behavioural evolution and ecology, and simultaneously underlines that so far, the lack of larval cases in the Permian and younger deposits may have purely resulted from taphonomic factors. As Recent species of marine caddisfly larvae are usually found in intertidal rock pools, they may be found in deeper, subtidal environments where they are swept by currents^45^,^46^. Although the possibility exists that the cases reported here may have experienced some transportation, the cases appear to be confined to the marine palaeoenvironment.

Interestingly, like the Recent examples from the Australasian region, the oldest larval cases reported here also come from southern, Gondwanan sector, of the Pangea palaeocontinent. Although there is still insufficient data from the fossil record^2^,^40^, based on the present discoveries, it is very possible that caddisflies whose larvae constructed cases in marine waters originated in southern hemisphere. However, the fossils documenting their potential spread across the southern Pangea marine waters have not been found yet. Thus, currently it is an enigma whether colonization of marine habitats by caddisfly ancestors consisted of a single event taking place in western part of the southern Pangea (South America), as supported by the present study, or multiple events taking place in different parts of the palaeocontinent in their long evolutionary history.

The fossiliferous deposits of the Campáleo outcrop, previously known from the excellently-preserved actinopterygian fishes^23^ and articulated hexactinellid sponges^26^, are now also known from the rich assemblages of fossils which, with some degree of confidence, represent the oldest caddisfly larval cases constructed in a marine palaeoenvironment of southern Pangea. It is thus the important, Late Palaeozoic Lagerstätte deposits which provide the unique insight into the origin and ecology of the oldest, case-making caddisflies.

## Methods

### Material and analysis

A total of 160 specimens of larval cases were collected and inspected using a binocular microscope and an environmental scanning electron microscope (ESEM) Philips XL30 at the Faculty of Earth Sciences, Sosnowiec, Poland. The specimens were inspected in an uncoated state using back-scattered (BSE) imaging in low vacuum conditions. The elemental composition was analysed by using the ESEM-coupled EDS detector. The material is housed at the Museu da Terra e da Vida, Centro Paleontológico (Cenpáleo) at the University of Contestado, Mafra, Brazil, under the registration numbers CPI-350–720 and CPE-3038–8000.

### Outcrop location

A location map of the outcrop studied is provided in Supplementary Fig. 1. The deposits of the outcrop studied are illustrated in Supplementary Fig. 2. Additional photos of the fossil larval cases are presented in Supplementary Fig. 3.

## Additional Information

**How to cite this article**: Mouro, L. D. *et al.* Larval cases of caddisfly (Insecta: Trichoptera) affinity in Early Permian marine environments of Gondwana. *Sci. Rep.* **6**, 19215; doi: 10.1038/srep19215 (2016).

## Supplementary Material

Supplementary Information
