# Supplementary material for: Larval cases of caddisfly (Insecta: Trichoptera) affinity in Early Permian marine environments of Gondwana
Source: Sci Rep. 2016 Jan 14;6:19215. doi: 10.1038/srep19215 (PMC4725916; doi:10.1038/srep19215)
Supplement: Supplementary Information [file srep19215-s1.doc]

**Supplementary Material**

**Larval cases of caddisfly (Insecta: Trichoptera) affinity in Early Permian**

**marine environments of Gondwana**

**Authors: Lucas D. Mouro^1,4^, Michał Zatoń^2*^, Antonio C.S. Fernandes^3^, and Breno L. Waichel^1^**

^1^Programa de Formação de Recursos Humanos, PFRH-PB 240,Universidade Federal de Santa Catarina, 88040-900, Brazil; ^2^Department of Palaeontology & Stratigraphy, Faculty of Earth Sciences, University of Silesia, 41200, Poland; ^3^Museu Nacional/Universidade Federal do Rio de Janeiro,20940-040, Brazil; ^4^Programa de Pós-Graduação em Geologia, Universidade Federal do Rio de Janeiro, 21.941-916, Brazil.

*Corresponding author: e-mail: mzaton@wnoz.us.edu.pl

**Supplementary Figure 1. Detail location of the Campáleo outcrop in the eastern part of the Paraná Basin, Brazil. The map was drawn on the basis of Waichel et al. (2013)^1^ using Corel Draw x5.**

**
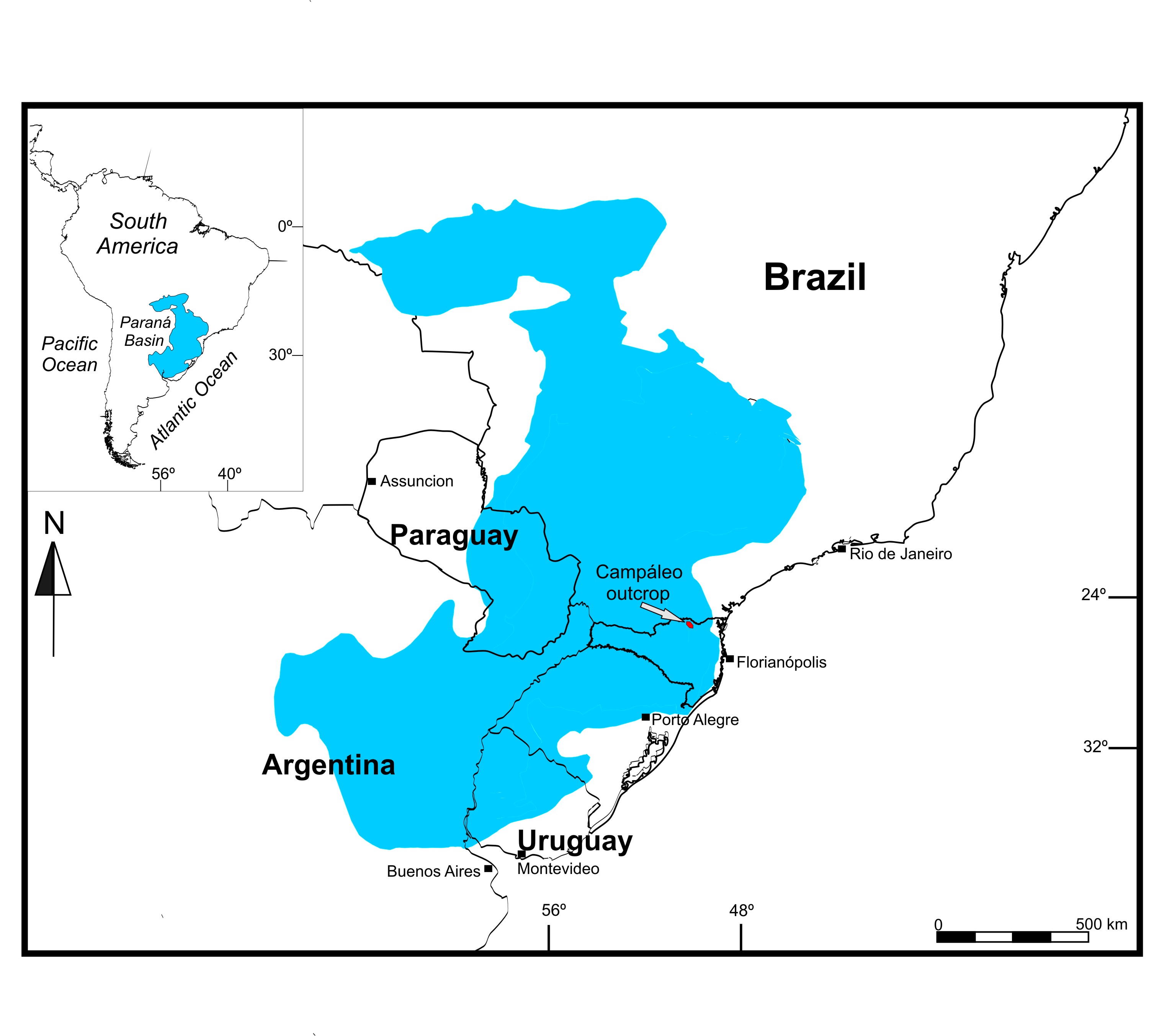
**

**Supplementary Figure 2. Lower Permian fossiliferous black shales containing the larval cases at the Campáleo outcrop. The frame is 1m^2^.**

**
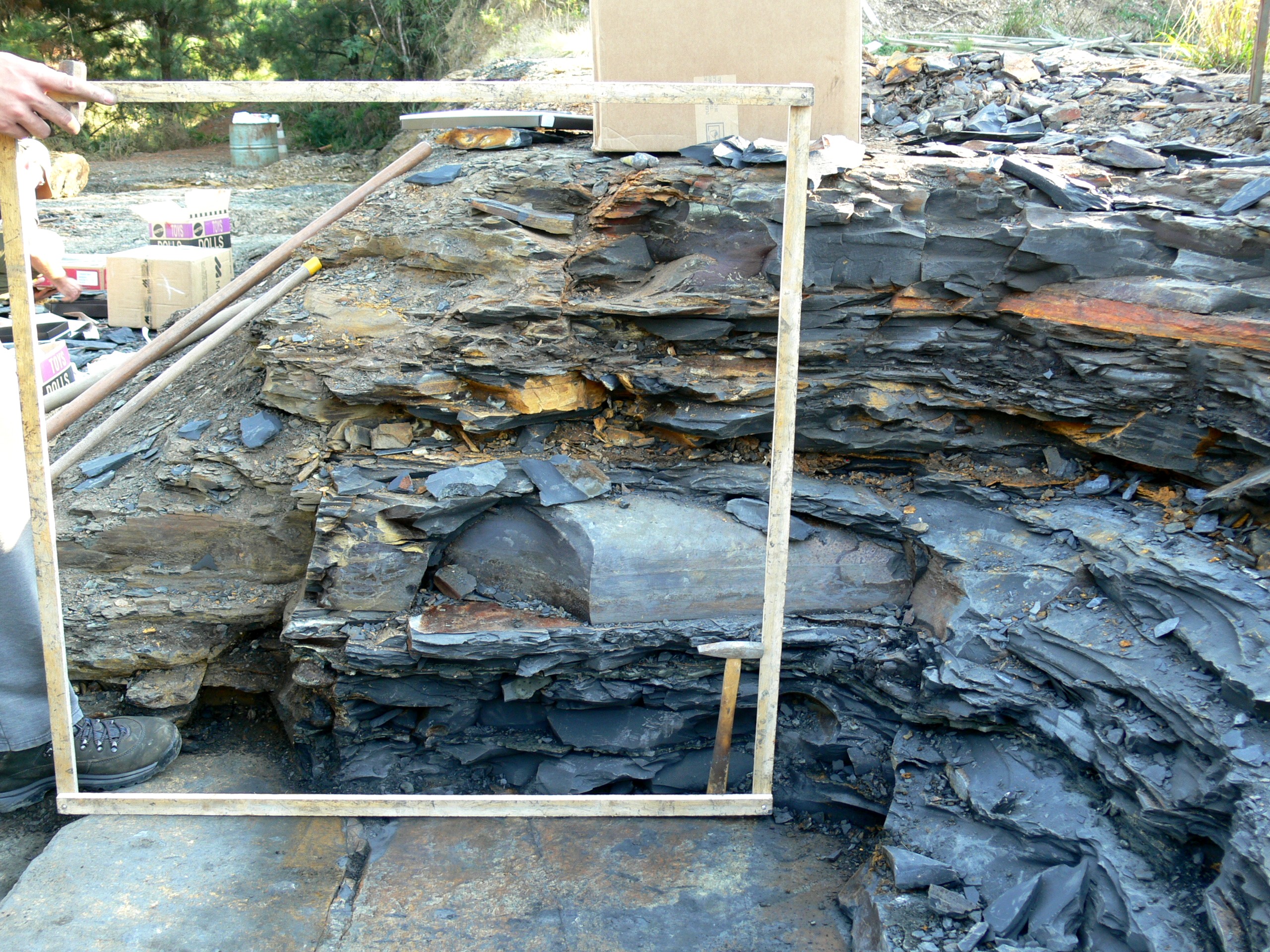
**

**Supplementary Figure 3. Additional examples of the fossils interpreted as caddisfly larval cases from the Lower Permian of the Campáleo outcrop, Brazil.** (a) A larval case on the bedding plane (white arrow) associated with abundant hectactinellid sponge skeletons (black arrows) at the Campáleo outcrop. (b) Disaggregated larval case in the form of isolated silk strips, CPE 6080. (c) Incomplete larval case surrounded by isolated silk strips, CPE 3090. (d-e) Incomplete larval cases; (d) CPE 6252, (e) CPE 5918. Scale bars 10 mm (a), 5 mm (b-e).

**
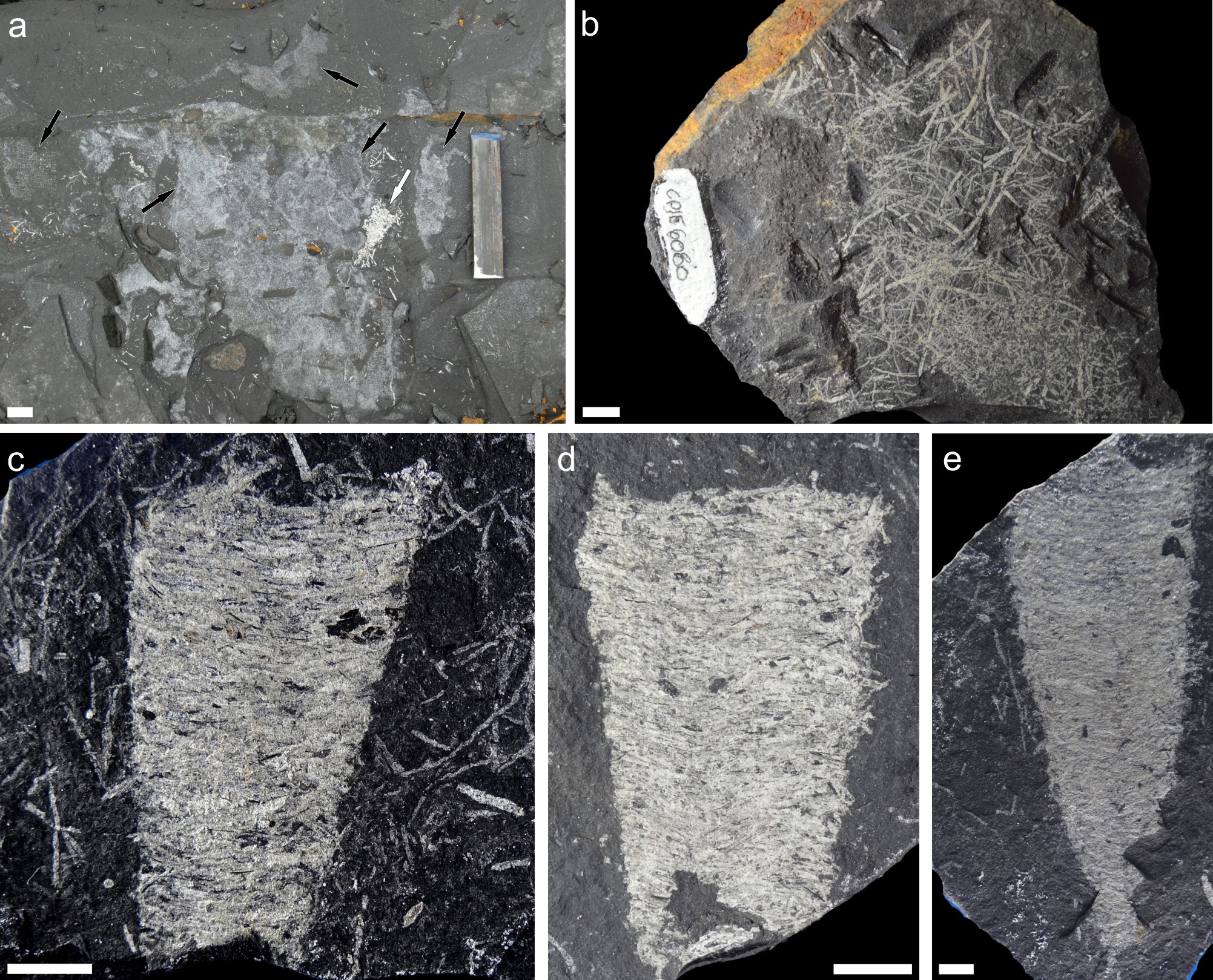
**

**References**

Waichel, B. L. et al. Lava tubes from the Paraná-Etendeka Continental Flood Basalt Province. Morphology and importance to emplacement models. *Journal of South American Earth Sciences

* **48**, 255–261 (2013).
